# Binding of DC-SIGN to the Hemagglutinin of Influenza A Viruses Supports Virus Replication in DC-SIGN Expressing Cells

**DOI:** 10.1371/journal.pone.0056164

**Published:** 2013-02-12

**Authors:** Marine L. B. Hillaire, Nella J. Nieuwkoop, Adrianus C. M. Boon, Gerrie de Mutsert, Stella E. Vogelzang-van Trierum, Ron A. M. Fouchier, Albert D. M. E. Osterhaus, Guus F. Rimmelzwaan

**Affiliations:** 1 Erasmus Medical Centre, Rotterdam, The Netherlands; 2 Viroclinics Biosciences BV, Rotterdam, The Netherlands; 3 Erasmus MC, Rotterdam, The Netherlands; Centre of Influenza Research, The University of Hong Kong, Hong Kong

## Abstract

Dendritic cells express lectins receptors, like DC-SIGN, which allow these cells to sense glycans that are present on various bacterial and viral pathogens. Interaction of DC-SIGN with carbohydrate moieties induces maturation of dendritic cells and promotes endocytosis of pathogens which is an important property of these professional antigen presenting cells. Uptake of pathogens by dendritic cells may lead to cross-presentation of antigens or infection of these cells, which ultimately results in activation of virus-specific T cells in draining lymph nodes. Little is known about the interaction of DC-SIGN with influenza A viruses. Here we show that a virus with a non-functional receptor binding site in its hemagglutinin, can replicate in cells expressing DC-SIGN. Also in the absence of sialic acids, which is the receptor for influenza A viruses, these viruses replicate in DC-SIGN expressing cells including human dendritic cells. Furthermore, the efficiency of DC-SIGN mediated infection is dependent on the extent of glycosylation of the viral hemagglutinin.

## Introduction

DC-SIGN (dendritic cell-specific intercellular adhesion molecule-3-grabbing nonintegrin) is a C-type lectin mainly present at the surface of dendritic cells (DC). DC are antigen presenting cells that play a key role in the induction of the adaptive immune responses. They are able to present antigens to T cells and induce their maturation. DC-SIGN signalling modulates the status of DC, triggers their maturation and promotes the adaptive immune response [1]. DC-SIGN belongs to the collectin family, and recognizes glycans of pathogens. For example ligands of DC-SIGN include bacteria and several viruses such as cytomegalovirus, Dengue virus, Ebola virus, hepatitis C virus, human immunodeficiency virus 1, SARS-coronavirus and West Nile virus [2], [3], [4], [5], [6], [7], [8], [9], [10]. Little is known about the interaction of DC-SIGN with influenza A viruses.

Influenza A viruses belong to the family of the Orthomyxoviridae. Their genome consists of eight negative stranded RNA segments which encode for eleven proteins. Two of these proteins, hemagglutinin (HA) and neuraminidase (NA), protrude from the viral envelope. They both recognize sialic acids on carbohydrate side chains of cellular glycoproteins and glycolipids. HA binds to sialic acids via its receptor binding site (RBS), which forms a pocket of highly conserved amino acids [11], [12], [13]. After binding to its receptor the virus is internalized via endocytosis. The low pH of the endosome causes HA to undergo conformational changes that lead to exposure of a fusion peptide and to fusion of the viral and endosomal membranes. The RNA segments of the virus are then delivered to the cytoplasm and transported to the nucleus, where replication is initiated. The new virions are assembled at the cell membrane and NA cleaves sialic acid at the cell membrane to allow the newly synthesized virions to detach from the cell.

Sialic acids are a critical factor for the tropism of the virus, because their type of linkage to a galactose residue determines whether they are recognized by specific viruses [14], [15]. Widely present in the avian gastrointestinal tract, α(2,3) linked sialic acids are preferably recognized by avian influenza A viruses [16], [17]. On the other hand, α(2,6) linked sialic acids are abundant in the human upper respiratory tract and preferably recognized by human influenza A viruses [18], [19], [20].

However, the binding of influenza A viruses to cells may not be restricted to recognition of sialic acids by the RBS of HA. It was found that lectin receptors can bind to influenza A viruses, suggesting that other means of virus attachment and subsequent entry could be involved [21], [22], [23], [24]. Cellular lectin receptors may recognize the glycans on HA, allow binding of the virus to the cells and its internalization. Thus, the extent of glycosylation of HA is likely to be important for the recognition of the virus by cellular lectins. Glycosylation is achieved by post translational modification of Asparagine residues of the NXS/T motif (X can be any amino acid except Proline). Numbers, types, and the positions of glycans vary for each virus, which might affect recognition of influenza viruses by lectin receptors such as DC-SIGN. However, the role of DC-SIGN in binding and entry of influenza viruses has been studied to a limited extent only [23], [25], [26]. Only a limited number of viruses has been investigated. Furthermore, although a correlation between the extent of glycosylation of viral envelope proteins and binding to DC-SIGN has been suggested, solid evidence for this is largely lacking. In addition, it is unclear if DC-SIGN mediated entry could support productive infection.

By introducing two mutations, L194AY195F, in the RBS of HA we created a mutant virus that was not able to bind to sialic acids. This mutant served to prove that DC-SIGN could recognize HA way and support virus replication. Then we selected nine H1N1 and H3N2 viruses to investigate whether the expression of DC-SIGN in cell lines and DC could support replication of these viruses in the absence of sialic acids. Furthermore, we genetically modified two viruses, A/Netherlands/602/09 (H1N1pdm09) and A/Netherlands/26/07 (H1N1), and inserted or deleted glycosylation sites on the head of HA and showed that binding efficiency of DC-SIGN to HA and subsequent infection rates are determined by the extent of glycosylation on the head of HA.

Finally we demonstrated that human DC can capture influenza A virus through interaction with DC-SIGN which leads to infection of these cells.

## Materials and Methods

### Cell lines

Madin-Darby Canine Kidney (MDCK) cells were cultured in Eagle's Minimum Essential medium; Vero cells in Dulbecco's Modified Eagle Medium. MDCK and Vero cells were stably transfected with a plasmid expressing human DC-SIGN (pcDNA3-DC-SIGN) that was kindly provided by Dr. V. Kewal Ramani. 4 µg of the plasmid was nucleofected into MDCK and Vero cells using the Amaxa® system (Lonza, Cologne, Germany). The next day the cells were washed and cultured in the presence of 0.25 mg/ml of G148. MDCK and Vero cells expressing DC-SIGN cells were isolated using CD209 MicroBeads (Miltneyi Biotec, Germany) following the manufacturer's instructions. DC-SIGN expression was monitored by flow-cytometry after incubation with anti CD209 antibody labeled with phycoerythrin (PE). After two passages MDCK DC-SIGN and Vero DC-SIGN cells were cultured in the presence of 0.25 mg/ml of G148. DC-SIGN expression was checked at each passage by flow cytometry.

DC-SIGN transgenic MDCK and Vero cells were generated after permission of the “Committee Genetic Modification” (COGEM), permit number 99-090.

### Viruses

An influenza virus with a deficient RBS was generated using reverse genetics. The amino acids at the positions 194 and 195 were targeted since they are crucial for receptor binding [11], [12], [27], [28].

To this end, the HA gene segment of influenza virus A/Puerto Rico/8/34 (A/PR/8/34) was modified by site directed mutagenesis (QuikChange multi site-directed mutagenesis kit, Stratagene, Leusden, Netherlands) to yield HA with L194A and Y195F amino acid substitutions [11], [12], [27], [28].

**Figure 1:**
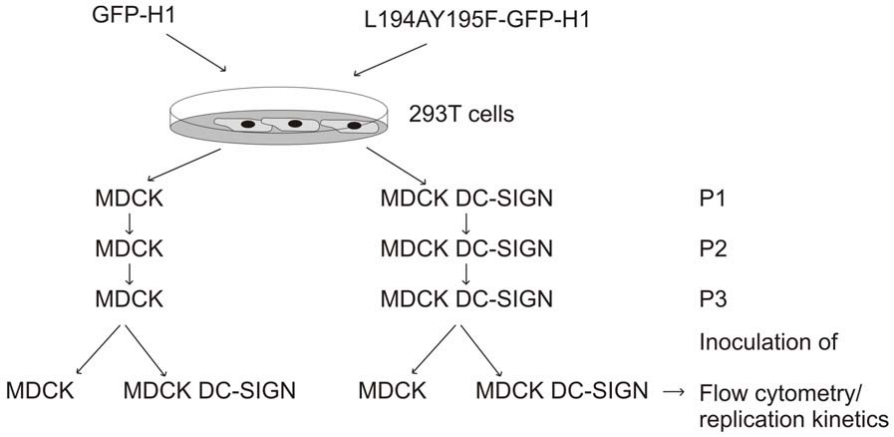
Schematic representation of the passage history and infection experiments with influenza viruses GFP-H1 and L194AY195F-GFP-H1.

Bidirectional reverse genetics plasmids [29], [30] containing wildtype (WT) or mutant HA were co-transfected into 293T cells with a plasmid encoding the NA gene segment of which the majority was replaced by the gene encoding Green fluorescent protein (GFP) [30] and plasmids encoding the remaining six gene segments of influenza virus A/PR/8/34. The supernatants were used to subsequently inoculate MDCK or MDCK-DC SIGN cells in the presence of neuraminidase from Vibrio cholerae (Sigma-Aldrich, Saint Louis, MO) (3.4 U/ml). The viruses, designated GFP-H1 and L194AY195F-GFP-H1 respectively, were used to subsequently inoculate MDCK or MDCK DC-SIGN cells (passage 2 and 3) (Figure 1).

Five H1N1 viruses (A/swine/Iowa/15/30, A/mallard/Netherlands/15/05, A/PR/8/34, A/USSR/90/77 and A/Netherlands/364/06) and four H3N2 viruses (A/swine/oedenrode/7C/96, A/Netherlands/35/93, A/Netherlands/312/03 and A/Netherlands/348/07) were selected and propagated in MDCK cells as described previously [31]. The culture supernatants of infected MDCK cells were clarified by low speed centrifugation, aliquoted and stored at −80°C until use. Infectious virus titers were determined as described previously [31].

A/Netherlands/602/09 and A/Netherlands/26/07 mutant viruses were made as previously described [32]. The HA gene segments of the two viruses were cloned into bidirectional reverse genetics plasmids, [29], . By site-directed mutagenesis (QuikChange multi site-directed mutagenesis kit, Stratagene, Leusden, Netherlands), the N-linked glycosylation sites were reciprocally exchanged to produce viruses that gained or lost one or more putative N-linked glycosylation sites. The plasmids encoding wild-type or mutant HA genes were co-transfected into 293T cells with plasmids encoding the remaining gene segments of back-bone strain A/PR/8/34 (H1N1) as described previously. Here we used A/Netherlands/26/07 and a mutant lacking one glycosylation site A/Netherlands/26/07-Δ125. We also used A/Netherlands/602/09, a mutant lacking one glycosylation site A/Netherlands/602/09-Δ276 and a mutant that contains three additional glycosylation sites that are present in A/Netherlands/26/07 (A/Netherlands/602/09-VN54 N125 N160).

### Replication curves and titration

Viruses L194AY195F-GFP-H1 and GFP-H1 passaged 2 or 3 times were used to inoculate MDCK or MDCK DC-SIGN cells at a MOI of 0.01. As the titer of passage 1 was too low for virus L194AY195F-GFP-H1, this passage was not used to determine multi-step replication kinetics. Culture supernatants were collected at 0, 6, 12, 24, 48 and 72 hours after inoculation and infectious virus titers were determined in MDCK or MDCK DC-SIGN cells as previously described [31].

### Flow cytometry

Viruses L194AY195F-GFP-H1 and GFP-H1 that were passaged 2 or 3 times were used to inoculate MDCK or MDCK DC-SIGN cells at a MOI of 0.01. GFP expression in infected cells was analyzed by flow cytometry at 24 and 48 hours post inoculation using a FACS calibur and Cell Quest Pro software (Becton and Dickinson).

### Infection assay

MDCK and Vero cells were treated with 3.4 U/ml neuraminidase from Vibrio cholerae (Sigma Aldrich, Zwijndrecht, The Netherlands) and GolgiStop (BD Biosciences, San Diego, CA) for 30 minutes to remove sialic acids from the cell surface. GolgiStop is a protein transport inhibitor and its use results in the accumulation of proteins in the Golgi complex.

Removal of sialic acids was confirmed by flow cytometry after staining with biotin-labeled Sambucus nigra (SNA) lectin (1/50 dilution) that binds to α(2,6) linked sialic acids and Maackia amurensis (MAA) lectin that binds to α(2,3) linked sialic acids for 30 minutes and subsequent staining with streptavidine labeled with fluorescein isothiocyanate (FITC) (Zebra Biosciences) following the manufacturer's instructions. We used a mixture of lectins SNA and MAA (both Sanbio BV, Uden, The Netherlands) to detect all sialic acid present on MDCK and Vero cells. This assay was performed to confirm that NA treatment was effective and to confirm the absence of both α2,3 and α2,6 sialosaccharides, which are both present on MDCK cells [30]

Untreated cells were used as positive controls. After inoculation with various viruses at a multiplicity of infection of 2 TCID_50_ per cell for one hour in the presence of 3.4 u/ml neuraminidase from vibrio cholerae and GolgiStop (BD Biosciences, San Diego, CA) the inoculum was aspirated and the cells were washed and incubated in culture medium for 16 hours. The cells were transferred to a 96-wells V-bottom plate and washed twice with PBS containing 2% Fetal Bovine Serum (P2F). They were stained for viability using AmCyan-labeled Live/dead staining (Invitrogen, Oregon, USA). After washing with P2F, the cells were fixed with 100 µl of cytofix (BD Biosciences, San Diego, CA) according to the manufacturer's recommendations. Subsequently, the cells were washed twice with cytoperm (BD Biosciences, San Diego, CA) and incubated with a monoclonal antibody specific for the viral nucleoprotein, labeled with FITC (DAKOCytomation, Glostrup, Denmark). After washing twice with P2F, the cells were analyzed by flow cytometry using the DIVA ® software. Each of the assays described above, was optimized and validated carefully using two different viruses A/Netherlands/364/06 and A/Netherlands/348/07. The reproducibility of the assays was confirmed by performing the assays at least three times. The final experiment with a large panel of viruses was performed in duplicate.

To confirm that entry was mediated by DC-SIGN, Vero and Vero DC-SIGN were treated with neuraminidase from vibrio cholerae for 30 minutes to remove sialic acids from the cell surface and incubated with or without 5 µg of antibodies to DC-SIGN (Abcam, Cambridge, UK) or an IgG2b isotype control (R&D systems, Minneapolis) or 40 mg/ml of mannose. These cells were subsequently inoculated with influenza virus (A/NL/312/03 or A/USSR/90/77). The percentage of infected cells compared to the positive control (untreated cells, still possessing of sialic acid) was assessed as described above. The infection assay experiments were performed in duplicate

### Dendritic cells

Peripheral blood mononuclear cells (PBMC) obtained from 3 healthy blood donors were isolated using Lymphoprep (Nycomed, Oslo, Norway) gradient centrifugation and cryopreserved at −135°C. Blood was obtained from Bloodbank Sanquin, region South West Netherlands, Rotterdam (Research permission number 10.084). Permission to use the PBMC for scientific research was obtained by informed consent. Dendritic cells were purified by MACS ® CD14 beads sorting (Miltenyi Biotec, Bergish Gladbach, Germany) and cultured for 7 days in the presence of 1000 u/mL GM-CSF and 200 u/mL IL-4. Then, DC were treated with 3.4 u/ml neuraminidase from vibrio cholerae (Sigma Aldrich, Zwijndrecht, The Netherlands) for 30 minutes to remove sialic acids present at the cell surface and incubated with 5 µg of monoclonal antibody to DC-SIGN (Abcam, Cambridge, UK) or an IgG2b isotype control (R&D systems, Minneapolis) to test if blocking DC-SIGN could inhibit infection of DC. They were subsequently inoculated overnight at a MOI of 3 with influenza viruses A/Netherlands/348/07 and A/Netherlands/312/03 in the presence of 3.4 u/ml neuraminidase from Vibrio cholerae (Sigma Aldrich, Zwijndrecht, The Netherlands). Subsequently the percentage of infected DC was assessed as described above. After optimization and validation, the final experiment was performed in triplicate.

## Results

### DC-SIGN expressing cell lines

**Figure 2:**
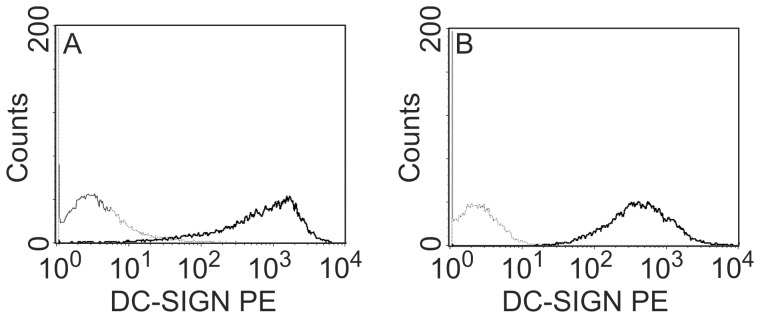
DC-SIGN expression in stably transfected MDCK DC-SIGN and Vero DC-SIGN cells. MDCK cells (A) and Vero cells (B) without (dotted line) and transfected with the gene encoding DC-SIGN (solid line) were analyzed for DC-SIGN expression after staining with a PE-labeled antibody to DC-SIGN and flow cytometry.

MDCK and Vero cells were stably transfected to express human DC-SIGN. The expression of DC-SIGN was assessed by flow-cytometry (figure 2). Typically >95% of cells expressed DC-SIGN.

### Replication kinetics

**Figure 3:**
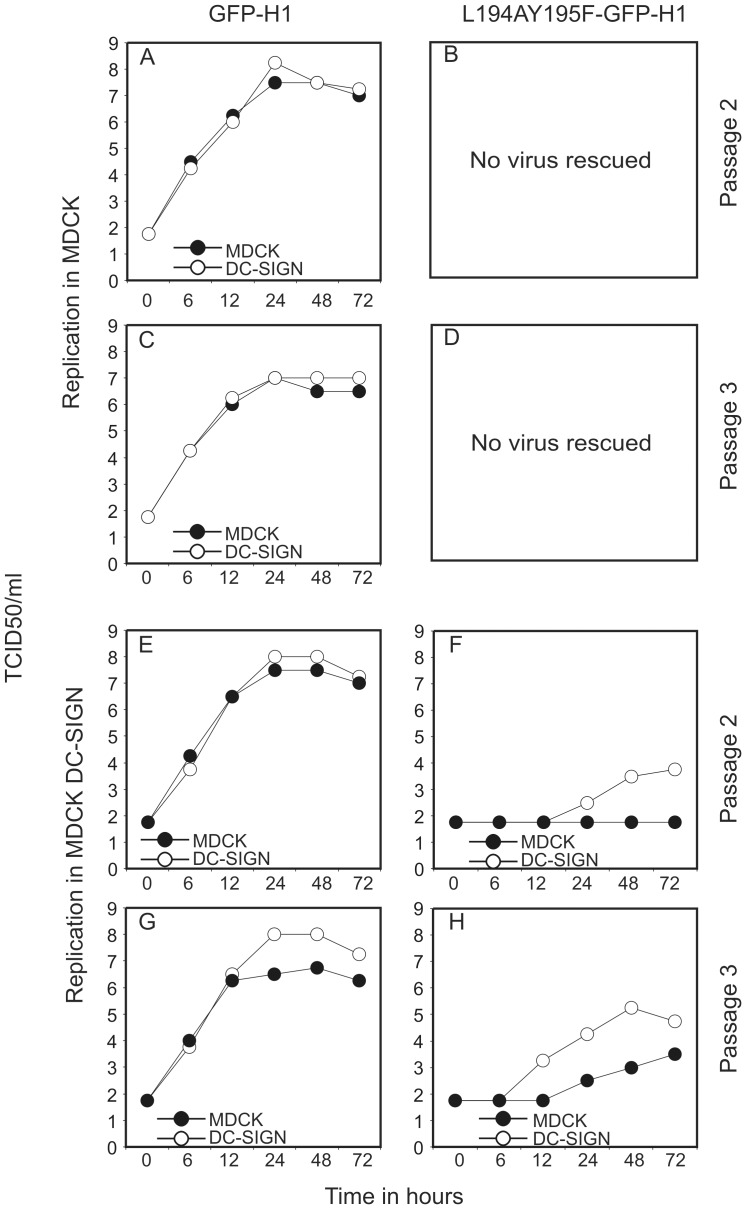
Replication kinetics of viruses GFP-H1 and L194AY195F-GFP-H1 in MDCK and DC-SIGN-expressing MDCK cells. After transfection of 293T cells with reverse genetics plasmids, culture supernatants of influenza viruses GFP-H1 (A, C, E, G) and L194AY195F-GFP-H1 (B, D, F, H) virus passaged in MDCK (A–D) and MDCK-DC-SIGN (E–H) cells were obtained and used to inoculate MDCK (solid symbols) or MDCK DC-SIGN cells (open symbols) at a moi of 0.01. At the indicated time points post inoculation culture supernatant were tested for the infectious virus titers to determine the replication kinetics. Virus L194AY195F-GFP-H1 could not be rescued in MDCK cells.

Viruses L194AY195F-GFP-H1 and GFP-H1 (passages 2 and 3) were used to inoculate MDCK or MDCK DC-SIGN cells at a MOI of 0.01 and culture supernatants were collected at various time points post inoculation (figure 1). The infectious virus titers were then determined in MDCK and MDCK DC-SIGN cells. The results are shown in figure 3. GFP-H1 virus replicated both in MDCK and MDCK DC-SIGN cells and after 24 hours the virus titers reached between 10^6.5^ and 10^8.25^ TCID50/ml after inoculation of MDCK or MDCK DC-SIGN cells for passage 2 and 3, respectively.

In contrast to virus GFP-H1, virus L194AY195F-GFP-H1 could not be rescued in MDCK cells (after five independent attempts) and no infectious virus was detectable after two subsequent passages in these cells (figure 3). However, upon passage in MDCK DC-SIGN cells, virus L194AY195F-GFP-H1 could readily be propagated although the titers that were reached were lower than for virus GFP-H1 (10^3.5^ and 10^5.25^ TCID50/ml 48 hours after inoculation of MDCK DC-SIGN cells for passage 2 and 3, respectively). After two passages in MDCK DC-SIGN cells, replication of virus L194AY195F-GFP-H1 in MDCK cells was undetectable, whereas after three passages a virus titer of 10^3.5^ TCID50/ml was reached in MDCK cells 72 hours post inoculation, which was almost 100-fold lower than the titer reached in MDCK DC-SIGN cells 48 hours post inoculation.

### Infection monitored by GFP expression

**Figure 4:**
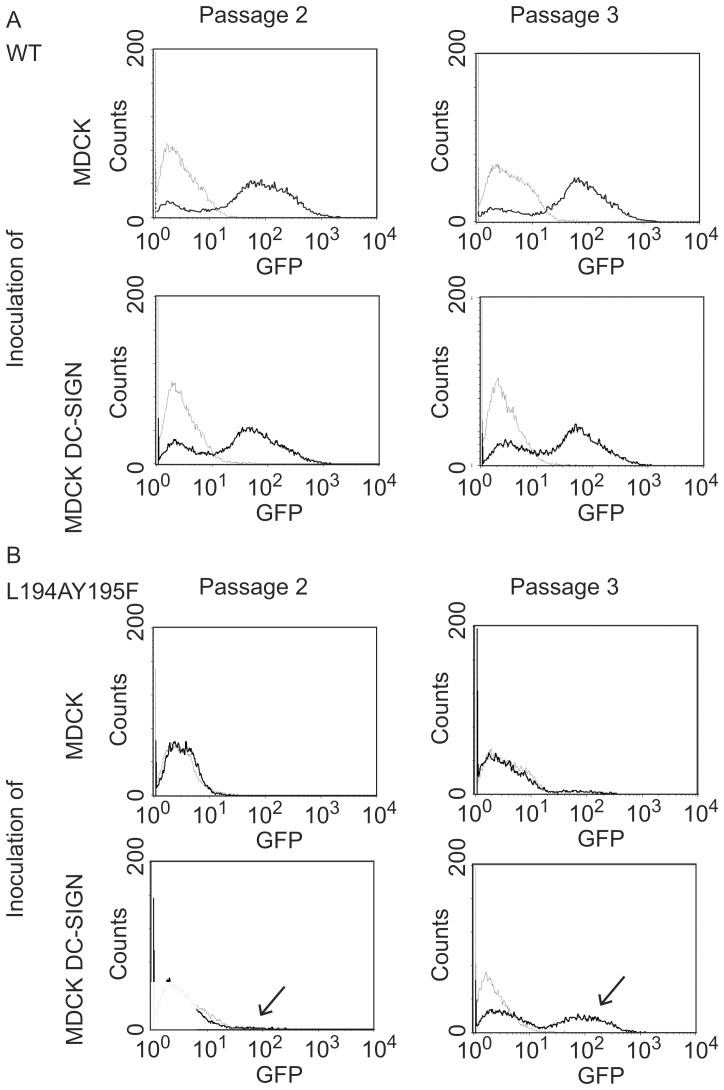
GFP expression after infection with L194AY195F-GFP and GFP-H1 in MDCK and DC-SIGN-expressing MDCK cells. MDCK and MDCK DC-SIGN cells were inoculated with influenza viruses GFP-H1 (A) and L194AY195F-GFP-H1 (B) at a MOI of 0.01 (solid lines). Both viruses were passaged in MDCK-DC-SIGN cells two or three times as indicated. Twenty-four hours post inoculation the cells were tested for GFP expression by flow cytometry. Uninfected cells were included as negative controls (dotted lines). Infection experiments with GFP-H1 virus passaged in MDCK cells essentially gave the same results as the virus passaged in MDCK DC-SIGN cells (data not shown).

Viruses L194AY195F-GFP-H1 and GFP-H1 (passages 2 and 3) were used to inoculate MDCK or MDCK DC-SIGN cells at a MOI of 0.01 and the expression of GFP was assessed by flow cytometry at 24 (figure 4) and 48 hours (data not shown) post inoculation.

The results confirmed that virus GFP-H1 with a functional HA molecule was able to infect MDCK and MDCK DC-SIGN cells regardless of the cell line that was used to produce this virus (figures 4A).

As shown in figure 4B, influenza A virus L194AY195F-GFP-H1 obtained after two or three passages in MDCK DC-SIGN cells infected a larger proportion of MDCK DC-SIGN cells than MDCK cells (arrows), which is in accordance with the differences in replication kinetics observed between the two cell lines.

### Influenza A viruses can infect cells via DC-SIGN in the absence of sialic acid

In order to remove sialic acids, the normal receptor for influenza A viruses, MDCK and Vero cells were treated with neuraminidase from Vibrio cholera. The success of this treatment was confirmed by flow cytometry using biotin-labeled lectins MAA and SNA (Figure S1). Untreated cells were used as positive controls for inoculation and the infection percentage of these cells were assessed. First we compared infection rates of untreated MDCK with MDCK DC-SIGN cells and Vero and Vero DC-SIGN cells. As expected the infection percentages did not differ significantly between cells that expressed DC-SIGN and those that did not. (Figure S2) (R^2^ values were of 0.7795 and 0.6416 for Vero and MDCK cells respectively). Since viruses displayed different infection rates, the infection rates were expressed relative to the positive control with sialic acids.

All nine viruses that were tested were able to infect MDCK, MDCK DC-SIGN, Vero and Vero DC-SIGN cells in the presence of sialic acids.

**Figure 5:**
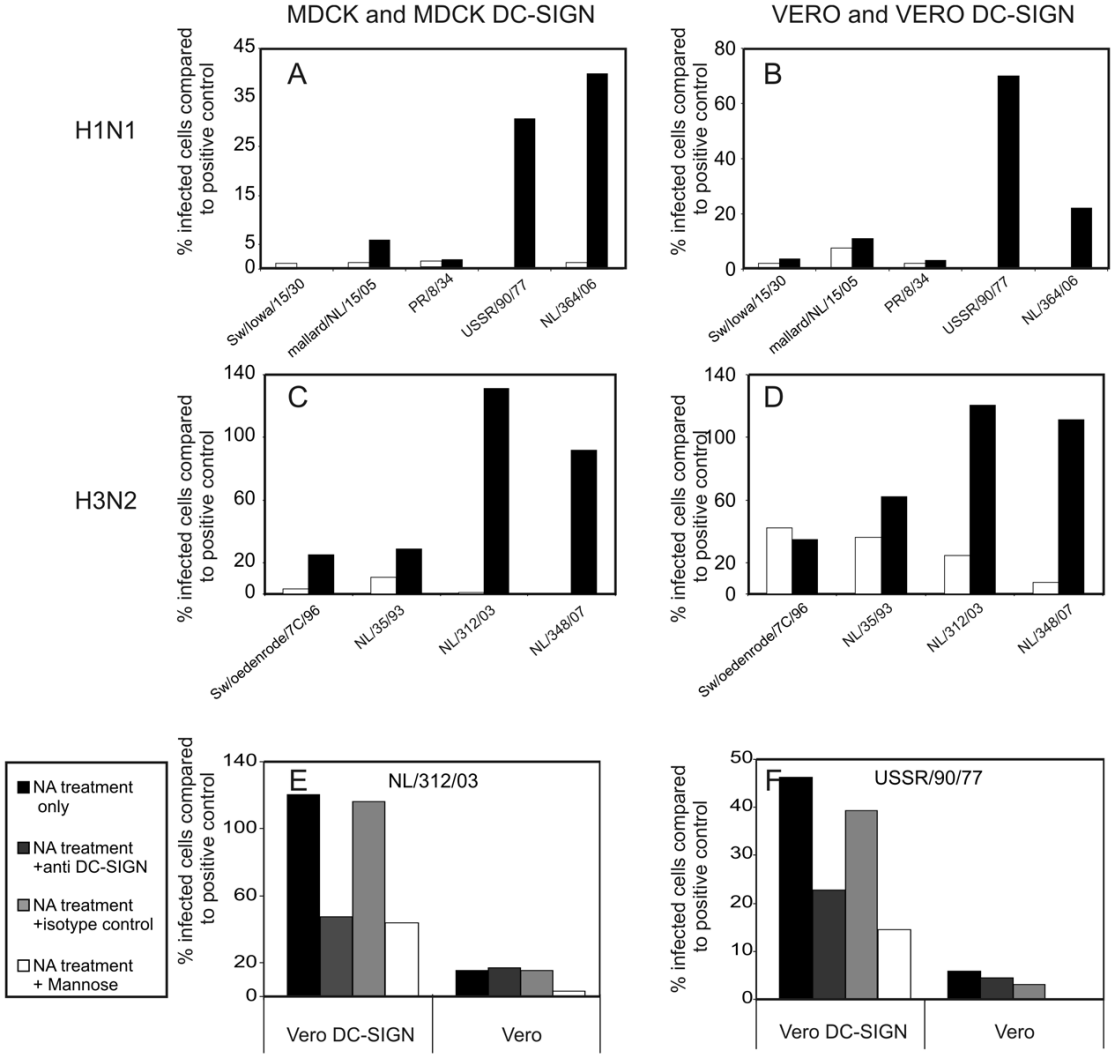
Expression of DC-SIGN supports replication of influenza A viruses in the absence of sialic acids. MDCK (A and C) and Vero cells (B and D) transfected with the DC-SIGN gene (black bars) or not (white bars), were treated with neuraminidase from vibrio cholerae and GolgiStop for 30 minutes to remove sialic acids from the cell surface. These cells were subsequently inoculated with five different A/H1N1 viruses (A and B) and four A/H3N2 viruses (C and D). The percentage infected cells relative to the untreated control cells, still possessing sialic acid, was assessed after detecting infected cells using a FITC-labelled antibody to the viral nucleoprotein and flow-cytometry. To confirm that the entry was mediated via DC-SIGN, Vero and Vero DC-SIGN were treated with neuraminidase from vibrio cholerae for 30 minutes to remove sialic acids from the cell surface and incubated with or without antibodies to DC-SIGN or an isotype control antibody as indicated (E and F). These cells were subsequently inoculated with influenza viruses. NL/312/03 and USSR/90/77. The percentage of infected cells compared to the positive control (untreated cells, still possessing sialic acid) was assessed as described above.

After removal of sialic acids from MDCK and Vero cells, the infection percentages relative to the untreated positive controls dropped considerably for most viruses. The mean relative number of infected cells was 13.6 and 2.0% for Vero cells and MDCK cells respectively (Figure 5). However, in the absence of sialic acids the expression of DC-SIGN supported the infection of MDCK cells and Vero cells by a number of influenza A viruses including two H1N1 viruses, A/USSR/90/77 and A/Netherlands/364/06, and four H3N2 viruses, A/swine/Oedenrode/7C/96, A/Netherlands/35/93, A/Netherlands/312/03 and A/Netherlands/348/07. Especially influenza A/H3N2 viruses A/Netherlands/312/03 and A/Netherlands/348/07 displayed high infection percentages, comparable to those of untreated control cells. A/H1N1 viruses A/swine/Iowa/15/30, A/mallard/Netherlands/15/05 and A/PuertoRico/8/34 displayed low infection percentages in MDCK-DC-SIGN cells devoid of sialic acids (0%, 1.9% and 2.6% respectively) and in Vero DC-SIGN cells (3.6%, 10.9% and 3.1% respectively) (figure 5 A and B). The differences observed between viruses may be explained by differences in the number of N-linked glycosylation sites present on HA (table 1). The number of putative N-linked glycosylation sites predicted with the online software NGlycNet correlated with DC-SIGN mediated infection of the cells. However, it remains unclear to which extent these glycosylation sites are in fact utilized.

**Table 1:** Number of putative N linked glycosylation sites present in HA1 and in HA2 of the viruses used in this study.

Virus	Number of putative glycosylation sites in		Predicted number of glycosylation sites in	
	HA1	HA2	HA1	HA2
swine/Iowa/15/30	1	5	1	4
mallard/NL/15/05	ND	ND	ND	ND
PuertoRico/8/34	1	6	4	5
USSR/90/77	4	6	3	5
NL/364/06	4	5	4	5
swine/oedenrode/7C/96	4	6	3	4
NL/35/93	2	5	1	4
NL/312/03	7	4	6	4
NL/348/07	7	4	5	3

The software NetNGlyc (http://www.cbs.dtu.dk/services/NetNGlyc/)was used to predict the number of glycosylation sites that will be utilized.

To confirm that entry of these viruses was mediated via DC-SIGN, Vero and Vero DC-SIGN cells were incubated with DC-SIGN blocking antibodies and subsequently infected with influenza viruses A/NL/312/03 and A/USSR/90/77. As shown in figure 5 E and F, the infection rates were reduced in the presence of these antibodies but not in the presence of control antibodies of the same isotype. Furthermore, also in presence of mannose, the entry was blocked, indicating that DC-SIGN was functional and able to bind to mannan.

### Influenza A viruses can infect DC via DC-SIGN

**Figure 6:**
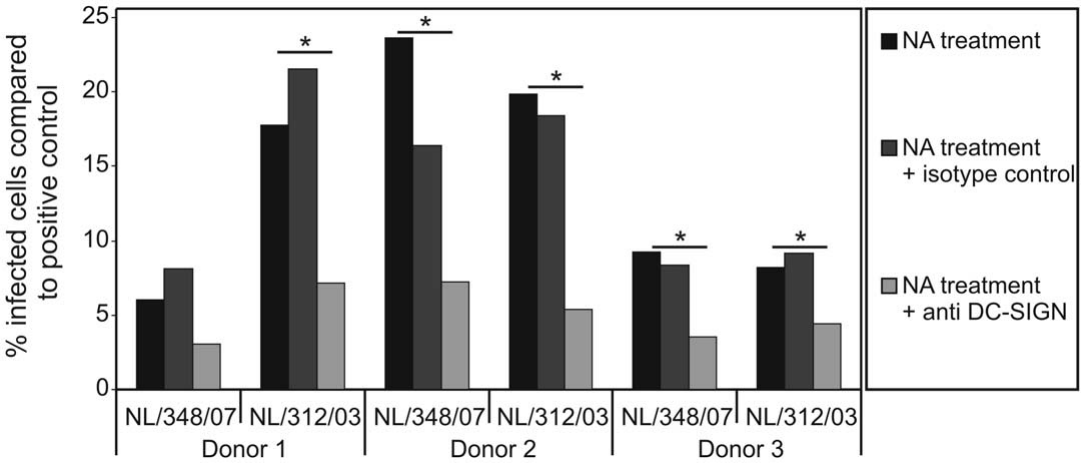
DC-SIGN expression on DC supports replication of influenza virus in absence of sialic acids. DC were treated with neuraminidase from vibrio cholerae for 30 minutes to remove sialic acids from the cell surface and incubated with or without antibodies to DC-SIGN or an isotype control antibody as indicated. These cells were subsequently inoculated with two A/H3N2 influenza viruses. The percentage of infected cells compared to the positive control (untreated cells, still possessing of sialic acid) was assessed as described above.

DC from three different healthy blood donors were isolated, treated with neuraminidase from Vibrio cholerae or not and subsequently inoculated with A/H3N2 viruses. Infection rates of DC obtained from different donors varied. Donor 1 displayed 6.6% and 20.3% infected cells after infection with A/Netherlands/348/07 and A/Netherlands/312/03 respectively. Donor 2 showed 5% and 9.6% infected cells after infection with A/Netherlands/348/07 and A/Netherlands/312/03 respectively and donor 3 displayed infection rates of 37% and 65% respectively. In the absence of sialic acids the infection percentages were reduced and ranged between 6 and 23% of those of untreated DC, depending on the virus and blood donor tested (figure 6). However, in presence of blocking anti-DC-SIGN antibodies this percentage further decreased significantly (p<0.05, Mann-Whitney test) whereas addition of control antibody did not have a significant effect on the infection percentages.

### Effect of glycosylation of HA on DC-SIGN mediated infection

**Figure 7:**
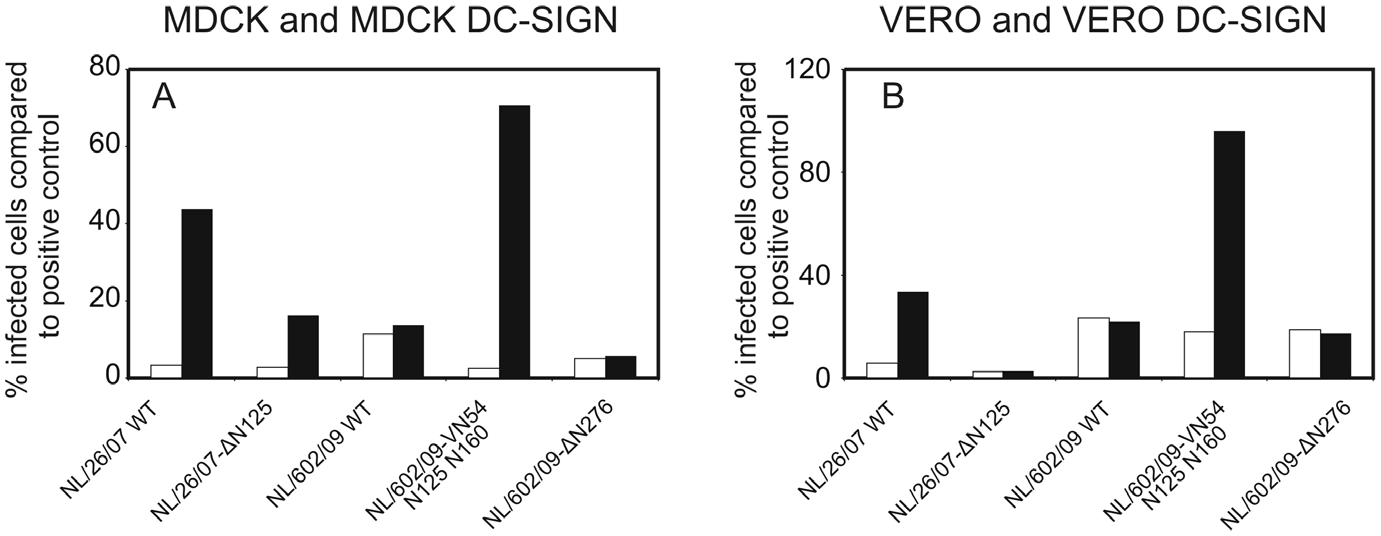
The number of glycosylation sites present on HA determines the virus infection rates in DC-SIGN expressing cells. MDCK (A) and Vero (B) cells, transfected with the DC-SIGN gene (black bars) or not (white bars) were treated with neuraminidase from vibrio cholerae and GolgiStop for 30 minutes to remove sialic acids from the cell surface. These cells were subsequently inoculated with A/Netherlands/26/07, A/Netherlands/26/07-Δ125, A/Netherlands/602/09, A/Netherlands/602/09-Δ276 or A/Netherlands/602/09-VN54 N125 N160. The percentage of infected cells relative to the positive control (untreated cells still possessing sialic acid) was assessed after detecting infected cells using a FITC-labeled antibody to the viral nucleoprotein and flow-cytometry.

Since DC-SIGN interacts with glycans present on membrane glycoproteins, we wished to investigate the effect of N-linked glycosylation of HA on binding to, and infection of, DC-SIGN expressing cells. To this end, we selected two viruses A/Netherlands/602/09 (H1N1pdm09) and A/Netherlands/26/07 (H1N1) and of which modification glycosylation sites on the head of the hemagglutinin were inserted or deleted. Influenza A virus A/Netherlands/26/07 infected DC-SIGN expressing Vero and MDCK cells, but hardly cells not expressing DC-SIGN (Figure 7). However, deletion of the glycosylation site at position 125 of HA (A/Netherlands/26/07-Δ125) severely impaired the capacity to infect cells in a DC-SIGN dependent fashion (figure 7). Influenza A virus A/Netherlands/602/09 displayed a low infection percentage in MDCK DC-SIGN (13.7%+/−0.2%) and Vero DC-SIGN cells (11.5%+/−0.3%), which was comparable to those of cells not expressing DC-SIGN. Influenza A virus A/Netherlands/602/09 lacking the N-linked glycosylation sites at position 276 (Δ276) infected cells, including DC-SIGN expressing cells, inefficiently. In contrast, insertion of three glycosylation sites in HA of A/Netherlands/602/09 (A/Netherlands/602/09-VN54 N125 N160), increased infection percentages of DC-SIGN expressing cells considerably compared to wild type virus and this virus infected DC-SIGN expressing cells almost as good as untreated cells with sialic acids on their surface (96%+/−1% and 70.6%+/−3% for MDCK DC-SIGN and Vero DC-SIGN, respectively).

## Discussion

In the present study we show that expression of DC-SIGN facilitates infection of cells by influenza A viruses independent of sialic acids, the natural receptor for these viruses, expressed on the target cell

First we generated a virus with mutations in its receptor binding site. It proved impossible to rescue virus L194AY195F-GFP-H1 in MDCK cells. However, using MDCK cells constitutively expressing DC-SIGN this virus was readily rescued. Furthermore, the mutant virus replicated better in DC-SIGN expressing cells than in normal MDCK cells. However, after three passages in MDCK DC-SIGN cells, virus L194AY195F-GFP-H1 displayed some replication in MDCK cells 48 hours post inoculation, although the kinetics of replication was delayed and the extent of replication reduced compared to that in MDCK cells expressing DC-SIGN. We determined the nucleotide sequence after each passage and did not find any sign of reversion of the RBS to the wild type sequence, although the emergence of minor variants that bind sialic acid cannot be excluded. Of interest, it has been recently demonstrated that influenza A viruses can enter CHO cells in a sialic acid independent way, although the exact mechanism remains elusive [34]. Alternatively, MDCK cells may support replication of the mutant virus by another unknown, but inefficient, way. Of note, we used A/PR/8/34 for generating a RBS deficient virus. However, in the absence of sialic acids, this virus replicated relatively poorly in DC-SIGN expressing MDCK and Vero cells, which may be related to the relative low number of N-Linked glycosylation sites in its HA and which may have reduced the window of opportunity to measure differences in infection rates in cells with and without DC-SIGN.

Next we explored an opposite approach, by removing the natural receptor for influenza A viruses from MDCK and Vero cells by treatment with neuraminidase and assessed the effect of DC-SIGN expression on infection ratesof nine different influenza A viruses of the H1N1 and H3N2 subtypes obtained from various species. Indeed for most viruses tested, the presence of DC-SIGN mediated virus entry and infection of the cells which can be explained by binding of DC-SIGN to glycans on the HA of these viruses, followed by endocytosis of the virus and fusion of the viral envelope with the endosomal membrane, the initial steps in the virus replication cycle. Of interest, the viruses that displayed efficient DC-SIGN mediated infection in the absence of sialic acids, had relatively large number of putative N-linked glycosylation sites on their HA

In order to confirm that indeed the interaction between DC-SIGN and glycans on HA are at the basis of the observed increased infection percentages in DC-SIGN expressing cells, we used genetically modified influenza A/H1N1 viruses, A/Netherlands/26/07 and A/Netherlands/602/09 (H1N1pdm09), of which N-linked glycosylation sites were reciprocally exchanged to produce viruses that gained or lost one or more putative N-linked glycosylation sites. In general, removal of N-linked glycosylation sites, reduced DC-SIGN mediated infection of MDCK and Vero cells, whereas addition of N-linked glycosylation sites increased infection of these cells (figure 7). Thus, DC-SIGN can act as an alternative receptor for influenza A viruses by binding to glycans present on HA and can initiate the virus replication cycle. Although, it has been suggested that the extent of glycosylation of HA determine the efficiency of recognition by DC-SIGN [25], we provide here for the first time solid evidence that this indeed is the case using a large panel of viruses and isogenic viruses with mutations in N-linked glycosylation sites only. Of interest, it was also suggested that H5N1 viruses can bind to DC-SIGN [23]. However, little infection of DC-SIGN expressing cells was observed after sialidase treatment of the cells. This might be explained by poor utilization of putative N-linked glycosylation sites in HA of the virus used, preventing binding to DC-SIGN. This was also observed after investigating the binding of H5N1 viruses to soluble C-type lectins such as porcine surfactant protein D [35].

Finally, we tested if DC, the cells of interest expressing DC-SIGN *in vivo* [36], [37] in addition to human alveolar macrophages [38], also can use this receptor for binding influenza A viruses. To this end, sialic acids were removed and it was shown that the infection of DC could be inhibited by a monoclonal antibody specific for DC-SIGN. Thus, DC can become infected in the absence of sialic acids after binding to glycans present on HA of influenza A virus as suggested previously for an H5N1 virus and DC cocultured with MDCK cells [23]. Most likely, the non-specific binding of glycosylated pathogens is a universal property of DC and may contribute to the induction of adaptive immune responses to these pathogens. Our findings may implicate that heavily glycosylated influenza A viruses more efficiently infect DC and subsequently may induce stronger immune responses than viruses that are less heavily glycosylated. Indeed the extent of glycosylation was shown to be inversely correlated with the virulence of influenza A viruses [39], [40], [41], which was attributed to increased sensitivity to the action of collectins, defence molecules of the innate immune system. Increased susceptibility to infection of DC may further blunt infection by heavily glycosylated influenza A viruses.

Collectively, we have shown that DC-SIGN can capture influenza A viruses and support infection of cells that express DC-SIGN. The efficiency of this process is dependent of the extent of glycosylation of the viral HA. Further research is required to relate the extent of DC-SIGN mediated infection of DC to the magnitude of the immune response to infection, the virus specific T cell response in particular.

## Supporting Information

Figure S1 Removal of sialic acids from cells by treatment with neuraminidase. Vero and MDCK cells were treated with neuraminidase from vibrio cholerae and GolgiStop for 30 minutes to remove sialic acids from the cell surface. After incubation with a mixture of biotin-labelled lectins SNA and MAA and subsequently with FITC-labelled streptavidin, the removal of sialic acids was confirmed by flow cytometry (in grey). The dotted line represents unstained cells and the black line cells represent the background staining of cells that were incubated with FITC-labelled streptavidin only.
(TIF)

Figure S2 Comparison of infection rates of MDCK and Vero cells with those expressing DC-SIGN after inoculation with 13 different viruses used in the present study at a multiplicity of infection of 2 TCID_50_ per cell. Each symbol represents an individual virus. These infection rates were used to calculate the “percentage of infection compared to positive control” showed in figure 5 and 6.
(TIF)

## References

[1] Svajger U, Anderluh M, Jeras M, Obermajer N (2010) C-type lectin DC-SIGN: an adhesion, signalling and antigen-uptake molecule that guides dendritic cells in immunity. Cell Signal 22: 1397–1405. 20363321 10.1016/j.cellsig.2010.03.018 PMC7127357

[2] Geijtenbeek TB, Kwon DS, Torensma R, van Vliet SJ, van Duijnhoven GC, et al (2000) DC-SIGN, a dendritic cell-specific HIV-1-binding protein that enhances trans-infection of T cells. Cell 100: 587–597. 10721995 10.1016/s0092-8674(00)80694-7

[3] Feinberg H, Mitchell DA, Drickamer K, Weis WI (2001) Structural basis for selective recognition of oligosaccharides by DC-SIGN and DC-SIGNR. Science 294: 2163–2166. 11739956 10.1126/science.1066371

[4] Yang ZY, Huang Y, Ganesh L, Leung K, Kong WP, et al (2004) pH-dependent entry of severe acute respiratory syndrome coronavirus is mediated by the spike glycoprotein and enhanced by dendritic cell transfer through DC-SIGN. J Virol 78: 5642–5650. 15140961 10.1128/JVI.78.11.5642-5650.2004 PMC415834

[5] Marzi A, Gramberg T, Simmons G, Moller P, Rennekamp AJ, et al (2004) DC-SIGN and DC-SIGNR interact with the glycoprotein of Marburg virus and the S protein of severe acute respiratory syndrome coronavirus. J Virol 78: 12090–12095. 15479853 10.1128/JVI.78.21.12090-12095.2004 PMC523257

[6] Halary F, Amara A, Lortat-Jacob H, Messerle M, Delaunay T, et al (2002) Human cytomegalovirus binding to DC-SIGN is required for dendritic cell infection and target cell trans-infection. Immunity 17: 653–664. 12433371 10.1016/s1074-7613(02)00447-8

[7] Pohlmann S, Zhang J, Baribaud F, Chen Z, Leslie GJ, et al (2003) Hepatitis C virus glycoproteins interact with DC-SIGN and DC-SIGNR. J Virol 77: 4070–4080. 12634366 10.1128/JVI.77.7.4070-4080.2003 PMC150620

[8] Alvarez CP, Lasala F, Carrillo J, Muniz O, Corbi AL, et al (2002) C-type lectins DC-SIGN and L-SIGN mediate cellular entry by Ebola virus in cis and in trans. J Virol 76: 6841–6844. 12050398 10.1128/JVI.76.13.6841-6844.2002 PMC136246

[9] Tassaneetrithep B, Burgess TH, Granelli-Piperno A, Trumpfheller C, Finke J, et al (2003) DC-SIGN (CD209) mediates dengue virus infection of human dendritic cells. J Exp Med 197: 823–829. 12682107 10.1084/jem.20021840 PMC2193896

[10] Martina BE, Koraka P, van den Doel P, Rimmelzwaan GF, Haagmans BL, et al (2008) DC-SIGN enhances infection of cells with glycosylated West Nile virus in vitro and virus replication in human dendritic cells induces production of IFN-alpha and TNF-alpha. Virus Res 135: 64–71. 18405996 10.1016/j.virusres.2008.02.008

[11] Skehel JJ, Wiley DC (2000) Receptor binding and membrane fusion in virus entry: the influenza hemagglutinin. Annu Rev Biochem 69: 531–569. 10966468 10.1146/annurev.biochem.69.1.531

[12] Wiley DC, Skehel JJ (1987) The structure and function of the hemagglutinin membrane glycoprotein of influenza virus. Annu Rev Biochem 56: 365–394. 3304138 10.1146/annurev.bi.56.070187.002053

[13] Wilson IA, Cox NJ (1990) Structural basis of immune recognition of influenza virus hemagglutinin. Annu Rev Immunol 8: 737–771. 2188678 10.1146/annurev.iy.08.040190.003513

[14] Connor RJ, Kawaoka Y, Webster RG, Paulson JC (1994) Receptor specificity in human, avian, and equine H2 and H3 influenza virus isolates. Virology 205: 17–23. 7975212 10.1006/viro.1994.1615

[15] Gambaryan AS, Tuzikov AB, Piskarev VE, Yamnikova SS, Lvov DK, et al (1997) Specification of receptor-binding phenotypes of influenza virus isolates from different hosts using synthetic sialylglycopolymers: non-egg-adapted human H1 and H3 influenza A and influenza B viruses share a common high binding affinity for 6′-sialyl(N-acetyllactosamine). Virology 232: 345–350. 9191848 10.1006/viro.1997.8572

[16] Suzuki Y (2005) Sialobiology of influenza: molecular mechanism of host range variation of influenza viruses. Biol Pharm Bull 28: 399–408. 15744059 10.1248/bpb.28.399

[17] Matrosovich MN, Gambaryan AS, Teneberg S, Piskarev VE, Yamnikova SS, et al (1997) Avian influenza A viruses differ from human viruses by recognition of sialyloligosaccharides and gangliosides and by a higher conservation of the HA receptor-binding site. Virology 233: 224–234. 9201232 10.1006/viro.1997.8580

[18] Thompson CI, Barclay WS, Zambon MC, Pickles RJ (2006) Infection of human airway epithelium by human and avian strains of influenza a virus. J Virol 80: 8060–8068. 16873262 10.1128/JVI.00384-06 PMC1563802

[19] Rogers GN, D'Souza BL (1989) Receptor binding properties of human and animal H1 influenza virus isolates. Virology 173: 317–322. 2815586 10.1016/0042-6822(89)90249-3

[20] van Riel D, Munster VJ, de Wit E, Rimmelzwaan GF, Fouchier RA, et al (2007) Human and avian influenza viruses target different cells in the lower respiratory tract of humans and other mammals. Am J Pathol 171: 1215–1223. 17717141 10.2353/ajpath.2007.070248 PMC1988871

[21] Reading PC, Miller JL, Anders EM (2000) Involvement of the mannose receptor in infection of macrophages by influenza virus. J Virol 74: 5190–5197. 10799594 10.1128/jvi.74.11.5190-5197.2000 PMC110872

[22] Upham JP, Pickett D, Irimura T, Anders EM, Reading PC (2010) Macrophage receptors for influenza A virus: role of the macrophage galactose-type lectin and mannose receptor in viral entry. J Virol 84: 3730–3737. 20106926 10.1128/JVI.02148-09 PMC2849513

[23] Wang SF, Huang JC, Lee YM, Liu SJ, Chan YJ, et al (2008) DC-SIGN mediates avian H5N1 influenza virus infection in cis and in trans. Biochem Biophys Res Commun 373: 561–566. 18593570 10.1016/j.bbrc.2008.06.078 PMC7092884

[24] Londrigan SL, Tate MD, Brooks AG, Reading PC (2012) Cell-surface receptors on macrophages and dendritic cells for attachment and entry of influenza virus. J Leukoc Biol 92: 97–106. 22124137 10.1189/jlb.1011492 PMC7166464

[25] Londrigan SL, Turville SG, Tate MD, Deng YM, Brooks AG, et al (2011) N-linked glycosylation facilitates sialic acid-independent attachment and entry of influenza A viruses into cells expressing DC-SIGN or L-SIGN. J Virol 85: 2990–3000. 21191006 10.1128/JVI.01705-10 PMC3067946

[26] Stray SJ, Cummings RD, Air GM (2000) Influenza virus infection of desialylated cells. Glycobiology 10: 649–658. 10910970 10.1093/glycob/10.7.649

[27] Martin J, Wharton SA, Lin YP, Takemoto DK, Skehel JJ, et al (1998) Studies of the binding properties of influenza hemagglutinin receptor-site mutants. Virology 241: 101–111. 9454721 10.1006/viro.1997.8958

[28] Gamblin SJ, Haire LF, Russell RJ, Stevens DJ, Xiao B, et al (2004) The structure and receptor binding properties of the 1918 influenza hemagglutinin. Science 303: 1838–1842. 14764886 10.1126/science.1093155

[29] de Wit E, Spronken MI, Bestebroer TM, Rimmelzwaan GF, Osterhaus AD, et al (2004) Efficient generation and growth of influenza virus A/PR/8/34 from eight cDNA fragments. Virus Res 103: 155–161. 15163504 10.1016/j.virusres.2004.02.028

[30] Rimmelzwaan GF, Nieuwkoop NJ, de Mutsert G, Boon AC, Kuiken T, et al (2007) Attachment of infectious influenza A viruses of various subtypes to live mammalian and avian cells as measured by flow cytometry. Virus Res 129: 175–181. 17714820 10.1016/j.virusres.2007.07.007

[31] Rimmelzwaan GF, Baars M, Claas EC, Osterhaus AD (1998) Comparison of RNA hybridization, hemagglutination assay, titration of infectious virus and immunofluorescence as methods for monitoring influenza virus replication in vitro. J Virol Methods 74: 57–66. 9763129 10.1016/s0166-0934(98)00071-8

[32] Hillaire ML, van Eijk M, Nieuwkoop NJ, Vogelzang-van Trierum SE, Fouchier RA, et al (2012) The number and position of N-linked glycosylation sites in the hemagglutinin determine differential recognition of seasonal and 2009 pandemic H1N1 influenza virus by porcine surfactant protein D. Virus Res 169: 301–305. 22921759 10.1016/j.virusres.2012.08.003

[33] Hoffmann E, Neumann G, Kawaoka Y, Hobom G, Webster RG (2000) A DNA transfection system for generation of influenza A virus from eight plasmids. Proc Natl Acad Sci U S A 97: 6108–6113. 10801978 10.1073/pnas.100133697 PMC18566

[34] de Vries E, de Vries RP, Wienholts MJ, Floris CE, Jacobs MS, et al (2012) Influenza A virus entry into cells lacking sialylated N-glycans. Proc Natl Acad Sci U S A 109: 7457–7462. 22529385 10.1073/pnas.1200987109 PMC3358892

[35] Hillaire ML, van Eijk M, van Trierum SE, van Riel D, Saelens X, et al (2011) Assessment of the Antiviral Properties of Recombinant Porcine SP-D against Various Influenza A Viruses In Vitro. PLoS One 6: e25005. 21935489 10.1371/journal.pone.0025005 PMC3173486

[36] Tailleux L, Schwartz O, Herrmann JL, Pivert E, Jackson M, et al (2003) DC-SIGN is the major Mycobacterium tuberculosis receptor on human dendritic cells. J Exp Med 197: 121–127. 12515819 10.1084/jem.20021468 PMC2193794

[37] Van Pottelberge GR, Bracke KR, Demedts IK, De Rijck K, Reinartz SM, et al (2010) Selective accumulation of langerhans-type dendritic cells in small airways of patients with COPD. Respir Res 11: 35. 20307269 10.1186/1465-9921-11-35 PMC2858735

[38] Soilleux EJ, Morris LS, Leslie G, Chehimi J, Luo Q, et al (2002) Constitutive and induced expression of DC-SIGN on dendritic cell and macrophage subpopulations in situ and in vitro. J Leukoc Biol 71: 445–457. 11867682

[39] Reading PC, Morey LS, Crouch EC, Anders EM (1997) Collectin-mediated antiviral host defense of the lung: evidence from influenza virus infection of mice. J Virol 71: 8204–8212. 9343171 10.1128/jvi.71.11.8204-8212.1997 PMC192277

[40] Tate MD, Brooks AG, Reading PC (2011) Specific Sites of N-Linked Glycosylation on the Hemagglutinin of H1N1 Subtype Influenza A Virus Determine Sensitivity to Inhibitors of the Innate Immune System and Virulence in Mice. J Immunol 187: 1884–1894. 21768397 10.4049/jimmunol.1100295

[41] Qi L, Kash JC, Dugan VG, Jagger BW, Lau YF, et al (2011) The ability of pandemic influenza virus hemagglutinins to induce lower respiratory pathology is associated with decreased surfactant protein D binding. Virology 412: 426–434. 21334038 10.1016/j.virol.2011.01.029 PMC3060949

