# Supplementary figures and images for: Binding of DC-SIGN to the Hemagglutinin of Influenza A Viruses Supports Virus Replication in DC-SIGN Expressing Cells
Source: PLoS One. 2013 Feb 12;8(2):e56164. doi: 10.1371/journal.pone.0056164 (PMC3570528; doi:10.1371/journal.pone.0056164)

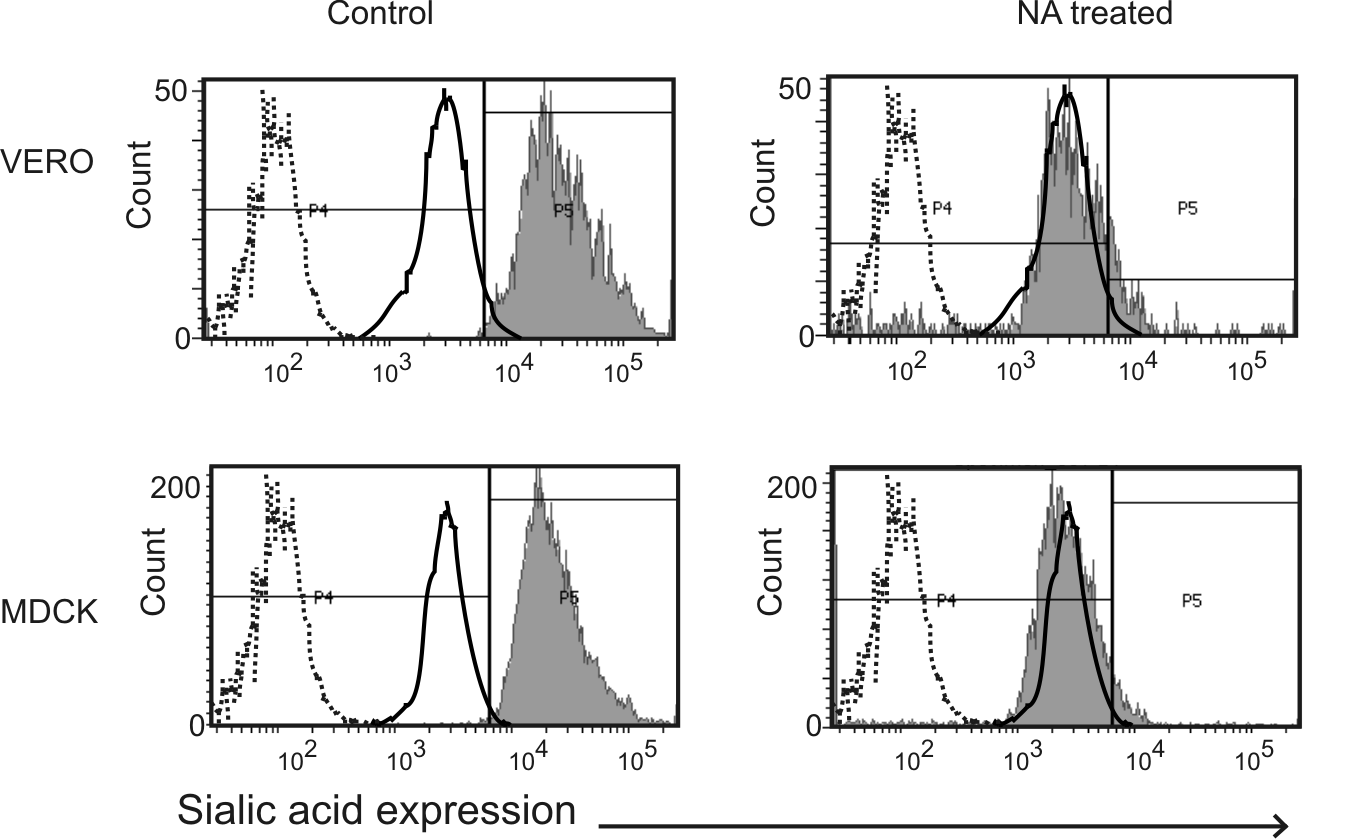

Supplement: Figure S1 — Removal of sialic acids from cells by treatment with neuraminidase. Vero and MDCK cells were treated with neuraminidase from vibrio cholerae and GolgiStop for 30 minutes to remove sialic acids from the cell surface. After incubation with a mixture of biotin-labelled lectins SNA and MAA and subsequently with FITC-labelled streptavidin, the removal of sialic acids was confirmed by flow cytometry (in grey). The dotted line represents unstained cells and the black line cells represent the background staining of cells that were incubated with FITC-labelled streptavidin only. (TIF) [file pone.0056164.s001.tif]

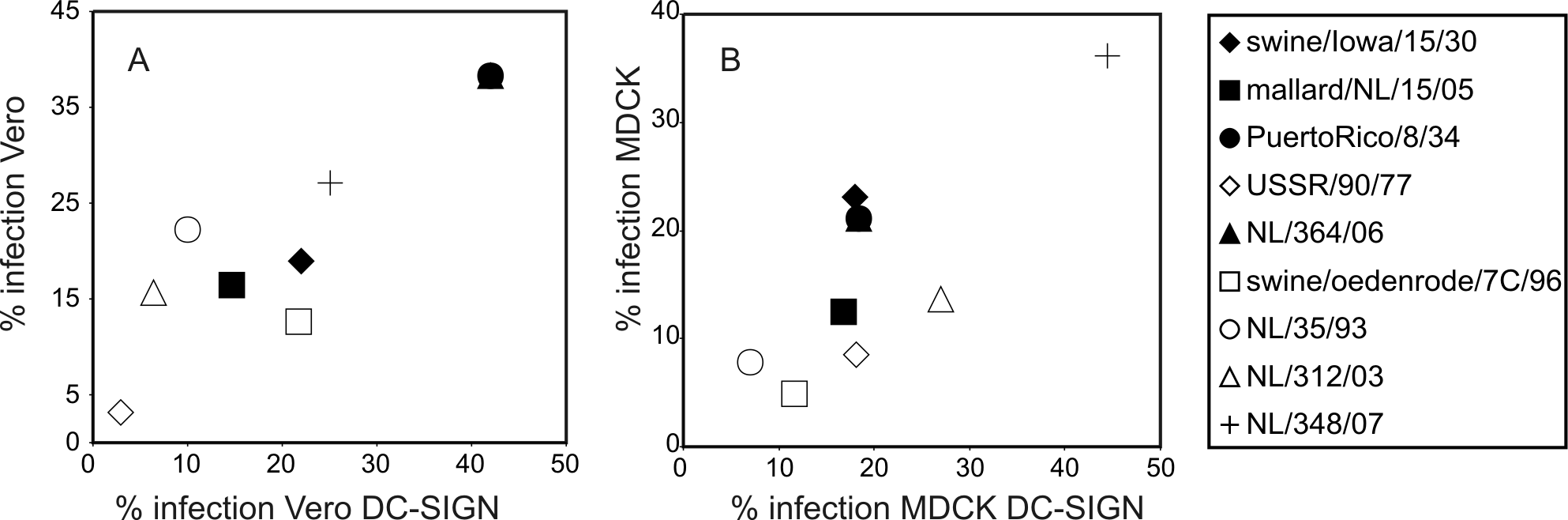

Supplement: Figure S2 — Comparison of infection rates of MDCK and Vero cells with those expressing DC-SIGN after inoculation with 13 different viruses used in the present study at a multiplicity of infection of 2 TCID50 per cell. Each symbol represents an individual virus. These infection rates were used to calculate the “percentage of infection compared to positive control” showed in figure 5 and 6. (TIF) [file pone.0056164.s002.tif]
